# Recent advances in managing and understanding Stevens-Johnson syndrome and toxic epidermal necrolysis

**DOI:** 10.12688/f1000research.24748.1

**Published:** 2020-06-16

**Authors:** Akito Hasegawa, Riichiro Abe

**Affiliations:** 1 Division of Dermatology, Niigata University Graduate School of Medical and Dental Sciences, Niigata, Japan

**Keywords:** Stevens-Johnson syndrome, toxic epidermal necrolysis, erythema multiforme, drug reaction, necroptosis

## Abstract

Stevens-Johnson syndrome (SJS) and toxic epidermal necrolysis (TEN) are life-threatening diseases characterized by detachment of the epidermis and mucous membrane. SJS/TEN are considered to be on the same spectrum of diseases with different severities. They are classified by the percentage of skin detachment area. SJS/TEN can also cause several complications in the liver, kidneys, and respiratory tract. The pathogenesis of SJS/TEN is still unclear. Although it is difficult to diagnose early stage SJS/TEN, biomarkers for diagnosis or severity prediction have not been well established. Furthermore, optimal therapeutic options for SJS/TEN are still controversial.

Several drugs, such as carbamazepine and allopurinol, are reported to have a strong relationship with a specific human leukocyte antigen (HLA) type. This relationship differs between different ethnicities. Recently, the usefulness of HLA screening before administering specific drugs to decrease the incidence of SJS/TEN has been investigated.

Skin detachment in SJS/TEN skin lesions is caused by extensive epidermal cell death, which has been considered to be apoptosis via the Fas-FasL pathway or perforin/granzyme pathway. We reported that necroptosis, i.e. programmed necrosis, also contributes to epidermal cell death. Annexin A1, released from monocytes, and its interaction with the formyl peptide receptor 1 induce necroptosis. Several diagnostic or prognostic biomarkers for SJS/TEN have been reported, such as CCL-27, IL-15, galectin-7, and RIP3.

Supportive care is recommended for the treatment of SJS/TEN. However, optimal therapeutic options such as systemic corticosteroids, intravenous immunoglobulin, cyclosporine, and TNF-α antagonists are still controversial. Recently, the beneficial effects of cyclosporine and TNF-α antagonists have been explored. In this review, we discuss recent advances in the pathophysiology and management of SJS/TEN.

## Introduction

Stevens-Johnson syndrome (SJS) and toxic epidermal necrolysis (TEN) are severe and life-threatening mucocutaneous reactions characterized by blisters and skin detachment. Drugs and infection, such as by *Mycoplasma* or the herpes simplex virus, are the main causes ^1^.

SJS/TEN are considered to be on the same spectrum of diseases with different severities. They are classified by the percentage of skin detachment area ( Table 1) ^2^. Although a study in the USA indicated that the incidence rate is 1.58 to 2.26 cases/million people, the overall incidence of SJS/TEN remains unclear. Contrary to its low incidence rate, the mortality rate is high (SJS: 4.8%, TEN: 14.8%) ^3^. Furthermore, even after recovery, sequelae such as blindness remain in some cases ^1^. Thus, patients with SJS/TEN should be accurately diagnosed, and appropriate treatment should commence as soon as possible. Therefore, a biomarker for early diagnosis and severity prediction is necessary. Further issues include the lack of evidence regarding the adequate management of SJS/TEN.

**Table 1.** Classification of SJS/TEN.

Diagnosis	Skin detachment area (%)
SJS	<10
SJS/TEN overlap	10–30
TEN	>30

SJS, Stevens-Johnson syndrome; TEN, toxic epidermal necrolysis

In this review, we describe recent advances in the research and management of SJS/TEN.

## Clinical features

The cutaneous symptoms for SJS/TEN are a painful erythematous rash, bullae, and erosion appearing on the face and trunk and spreading to the extremities. The early skin lesions appear as round lesions with only two nonpalpable zones with an indistinct border and are called “atypical targets”. Skin lesions typically test positive for the Nikolsky sign, which manifests with skin erosion upon gentle pressure ^4^. Malaise, fever, and upper respiratory tract symptoms often precede the onset of the skin rash by a few days. Almost all patients with SJS/TEN develop mucosal involvement of the eyes, mouth, and genitalia ^5^. The involvement of the eyes often relates to sequelae such as dry eyes, visual acuity, conjunctivitis, corneal erosions, and trichiasis. In severe cases, ocular sequelae can reach as far as blindness.

The severity-of-illness score for TEN (SCORTEN) is widely used to predict mortality for SJS/TEN ^6^. SCORTEN should be assessed within the first 24 hours after admission and again on day 3. SCORTEN is based on seven independent risk factors ( Table 2). The more risk factors that are present, the higher the mortality rate ( Table 3).

**Table 2.** Risk factors for SCORTEN.

Age over 40 years
Heart rate >120 beats per minute
Presence of cancer or hematologic malignancy
Epidermal detachment area involving body surface area >10%
Blood urea nitrogen >28 mg/dL (10 mmol/L)
Blood glucose >252 mg/dL (14 mmol/L)
Bicarbonate <20 mEq/L

SCORTEN, Score of Toxic Epidermal Necrosis

**Table 3.** Mortality rate in SCORTEN.

Number of risk factors	Mortality rate (%)
0–1	3.2
2	12.1
3	35.3
4	58.3
≥5	90

SCORTEN, Score of Toxic Epidermal Necrosis

SJS/TEN are mainly drug-induced diseases. The most frequently causative drugs include antibiotics, allopurinol, non-steroidal anti-inflammatory drugs, and antiepileptic drugs ( Table 4) ^7^.

**Table 4.** Medications associated with high risk of SJS/TEN.

Nevirapine
Lamotrigine
Carbamazepine
Phenytoin
Cotrimoxazole and other anti-infective sulfonamides
Sulfasalazine
Allopurinol
Oxicam/NSAIDs

NSAID, non-steroidal anti-inflammatory drug; SJS, Stevens-Johnson syndrome; TEN, toxic epidermal necrolysis

## Genetic factors

There is increasing evidence of a genetic contribution to the incidence of cutaneous adverse reactions. In 2004, Chung *et al.* reported on a strong relationship between human leukocyte antigen (HLA)-B*15:02 and carbamazepine (CBZ)-induced SJS/TEN in a Han Chinese population ^8^. HLA alleles are divided into class I and class II, and they are specialized to present antigenic peptides to T cells, resulting in the activation of the immune response. In this study, 44 patients with CBZ-induced SJS/TEN were included, and all patients had the HLA-B*15:02 allele (100%). Following this, similar studies reported the relationship between CBZ-induced SJS/TEN and the HLA-B*15:02 allele in Asian populations including those in China, Thailand, Malaysia, and India ^9– 20^.

The relationship between SJS/TEN and HLA-B*15:02 has also been demonstrated in aromatic antiepileptic drugs other than CBZ. Although the incidence was lower than that seen with CBZ, HLA-*15:02 showed a strong association with phenytoin-, lamotrigine-, and oxcarbazepine-induced SJS/TEN ^11, 21– 25^. Conversely, there was no association between CBZ-induced SJS/TEN and HLA-B*15:02 in Japanese, Korean, and European populations ^26– 32^.

Ozeki *et al.* discovered that HLA-A*31:01 is also associated with CBZ-induced SJS/TEN ^33^. HLA-A*31:01 revealed a relationship with CBZ-induced SJS/TEN not only in Japanese but also in Korean and European populations ^14, 32, 34, 35^. Although the majority of CBZ-induced SJS/TEN is associated with HLA-B*15:02 in Asian populations, the association with HLA-A*31:01 is shown in multiethnic populations. Thus, the HLA association in SJS/TEN is different among different ethnicities.

In 2008, the US Food and Drug Administration released a recommendation to perform HLA-B*15:02 genotyping before administering CBZ ^36^. In Taiwan, it is reported that HLA-B*15:02 screening is strongly associated with a decrease in the incidence of CBZ-induced SJS/TEN ^37^.

As well as antiepileptic drugs, several other drugs, such as allopurinol and abacavir, have been reported to have HLA associations. Allopurinol is an anti-hyperuricemia drug which is a major cause of SJS/TEN. The relationship between HLA-B*58:01 and allopurinol-induced SJS/TEN has been reported in many ethnicities, including in Taiwanese, Japanese, Korean, Thai, and European individuals ^26, 28, 30, 38– 45^. Therefore, these data suggested that HLA-B*58:01 genotyping may be useful to prevent allopurinol-induced SJS/TEN.

Cost-effectiveness analysis of HLA-B*58:01 screening in Taiwan suggested a cost-saving effect in preventing allopurinol-induced SJS/TEN ^46^. In a US study, it was suggested that testing for HLA-B*5801 prior to allopurinol initiation is cost effective for Asians and African Americans but not for Caucasians or Hispanics ^47^.

Abacavir, a nucleoside reverse transcriptase inhibitor used to treat HIV infection, is reported to induce SJS/TEN in patients carrying HLA-B*57:01 ^48– 52^. In 2008, HLA-B*57:01 screening was added to clinical care guidelines to reduce the risk of hypersensitivity reaction from abacavir ^53^. The frequency of HLA-B*57:01 screening then increased steadily, and the incidence of abacavir-induced SJS/TEN was decreased ^54^. However, many patients have not undergone HLA-B*57:01 screening. The expansion of HLA*B-57:01 screening is expected to reduce the incidence of abacavir-induced SJS/TEN.

Cytochrome P (CYP) is also an important genetic factor. CYPs are involved in drug metabolism. CYP450 genes have 57 variants, and each variant shows functional differences. Patients whose drug metabolism is slow because of CYP450 variants have a high risk of developing adverse drug reactions ^55^. Chung *et al.* discovered specific genetic factors associated with phenytoin-induced SJS/TEN ^56^. In this study, 16 significant single nucleotide polymorphisms in CYP2C9 were identified. Patients with phenytoin-induced SJS/TEN who had CYP2C9*3 showed a delayed clearance of phenytoin, resulting in increased disease severity.

## Pathogenesis and diagnostic biomarkers

### Immunopathogenesis

**Figure 1.**
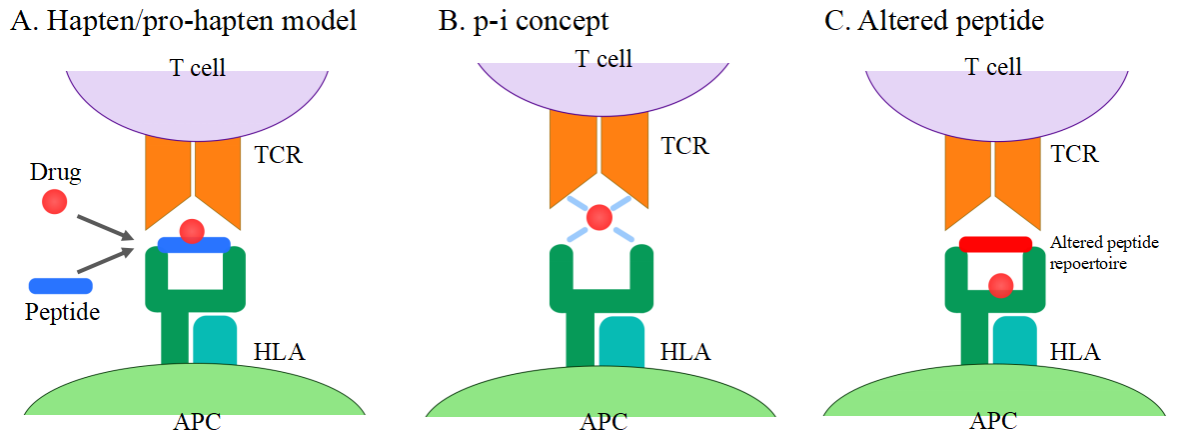
Models of T cell activation in Stevens-Johnson syndrome/toxic epidermal necrolysis. ( **A**) Hapten/pro-hapten model: drugs or drug metabolites form a complex with carrier proteins and are presented as haptenated peptides in the peptide-binding groove of the HLA molecules. ( **B**) p-i concept: drugs directly bind to HLA and TCR non-covalently. ( **C**) Altered peptide model: drugs bind to the peptide-binding groove of HLA, resulting in the alteration of the HLA-binding peptide repertoire. APC, antigen-presenting cell; HLA, human leukocyte antigen; TCR, T cell receptor.

SJS/TEN is traditionally thought to be a T-cell-mediated disorder. T cells are activated by binding of drugs to T cell receptors (TCRs) from antigen-presenting cells (APCs). There are currently three hypotheses on T cell activation ^57– 59^ ( Figure 1): (1) the hapten/pro-hapten model, (2) the pharmacological interaction (p-i) concept, and (3) the altered peptide model. The majority of drugs and their metabolites are pro-haptens and do not act as haptens themselves. They acquire the immunogenicity by covalently binding to carrier proteins (hapten antigen). Hapten antigens form a complex with HLA in APCs and are recognized by TCRs. This stimulation triggers the drug-specific T cell activation. In this model, antigenic drugs are covalently bound to peptides presented by HLA molecules to TCRs ^60– 63^. However, some drugs can non-covalently bind directly to HLA and/or TCRs. This type of binding is termed the p-i concept. CBZ, lamotrigine, sulfamethoxazole, and celecoxib are known to fit this model ^64– 68^. In general, HLA polymorphisms are dependent on the antigen-binding cleft. It has been reported that unmodified abacavir binds to the antigen-binding cleft lying in the bottom of HLA-B*57:01 and changes the shape and chemistry of the antigen-binding cleft, altering the repertoire of endogenous peptides that can bind HLA-B*57:01 (altered peptide) ^69, 70^. The TCR profile is also associated with the development of SJS/TEN. Ko *et al.* identified the VB-11-ISGSY clonotype in 84% of patients with CBZ-associated SJS/TEN ^71, 72^. This clonotype was not present in CBZ-tolerant patients. The clonotype specificity is also reported in oxypurinol-induced SJS/TEN ^73^. Recently, Pan *et al.* investigated the TCR repertoire through next-generation sequencing and identified a public αβTCR from the cytotoxic T cells of patients with CBZ-induced SJS/TEN. This public αβTCR can bind with CBZ and mediate an immune response ^74^.

In the early stages of the disease, cytotoxic CD8 ^+^ T cells mainly infiltrate blister fluid and the epidermis, and CD4 ^+^ T cells mostly infiltrate the dermis ^75, 76^. Monocytes are present in the epidermis of TEN patients. In the later stages, lymphocytes are decreased and an increased number of monocytes is observed. Tohyama *et al.* reported that monocytes play an important role in epidermal damage, probably by enhancing the cytotoxicity of CD8 ^+^ T cells ^77^. In the serum and blister fluid of SJS/TEN patients, increased levels of soluble IL-2 receptors were observed ^78^. Soluble IL-2 receptors are a marker for activated T cells, indicating the importance of activated cytotoxic CD8 ^+^ T cells in the pathogenesis of SJS/TEN.

### Keratinocyte death

The epidermal damage in the skin lesions of SJS/TEN patients is considered to be of apoptotic origin ^79^. Apoptosis is induced by cytotoxic CD8 ^+^ T cells through the Fas-Fas ligand (FasL) pathway or the perforin/granzyme pathway ^80^.

Cytotoxic CD8 ^+^ T cells and natural killer (NK) cells produce FasL, which binds Fas on target cells. Recognition of FasL causes activation of the caspase cascade and the resulting cells undergo apoptosis ^80^. Under normal conditions, Fas is present on the surface of keratinocytes and FasL is expressed intracellularly. FasL is transported to the cell surface when the cell needs to self-destruct ^81^. Viard *et al.* demonstrated that the cell surface of keratinocytes of TEN patients has FasL on it but not the keratinocytes of patients with maculopapular drug reactions ^82^. In addition, high levels of soluble FasL (sFasL) were found in the serum of TEN patients. sFasL also has the potential to mediate apoptosis ^83^.

We showed that FasL serum levels increased in patients with TEN ^84, 85^. This study revealed that sFasL was produced by peripheral blood mononuclear cells (PBMCs) when the causative drugs were added. sFasL released from PBMCs binds to Fas expressed on keratinocytes to cause apoptosis. This study suggested that elevated levels of serum sFasL may be a useful diagnostic marker for SJS/TEN. However, a correlation between sFasL levels and disease severity has not been established ^84, 86^.

Nassif *et al.* emphasized the importance of the perforin/granzyme pathway ^87, 88^. Upon recognition of a target cell, the cytotoxic CD8 ^+^ T cell releases perforin and granzyme B ^80^. This study revealed that mononuclear cells in the blister fluid of TEN patients have cytotoxic effects in the presence of a causative drug. This cytotoxicity is blocked by the perforin/granzyme pathway inhibitor. These findings suggest that the perforin/granzyme pathway causes epidermal damage in the skin lesions of SJS/TEN ^87, 88^.

In 2008, Chung *et al.* demonstrated the cytotoxic effect of granulysin in SJS/TEN ^89^. Granulysin is a pro-apoptotic protein which permits cell-mediated cytotoxicity without direct cell-to-cell contact. In SJS/TEN blisters, high levels of granulysin are detected. Granulysin is released from blister cells in skin lesions of SJS/TEN including cytotoxic CD8 ^+^ T cell and NK cells. The severity of the cutaneous lesions correlated with serum granulysin levels. We also reported granulysin as an early diagnostic marker ^90^. However, serum granulysin levels are also elevated in patients with drug-induced hypersensitivity syndrome/drug reactions with eosinophilia and systemic symptoms, which are other types of severe cutaneous adverse drug reaction characterized by a viral infection ^91^. Therefore, it is difficult to use granulysin as an SJS/TEN-specific biomarker.

**Figure 2.**
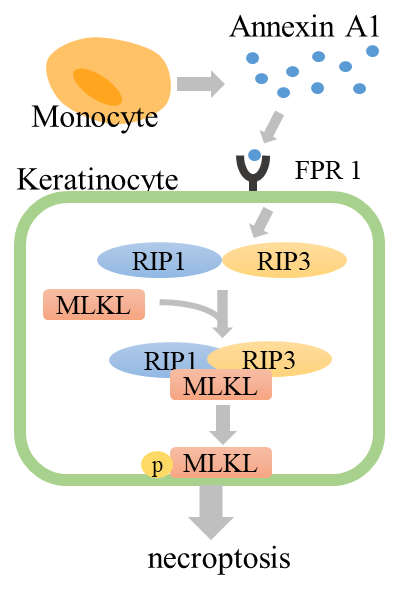
Necroptosis pathway in Stevens-Johnson syndrome/toxic epidermal necrolysis. Drug-stimulated monocytes secrete annexin A1. Annexin A1 binds to FPR1, RIP1 and RIP3 form the necrosome, and MLKL is phosphorylated by RIP3. Phosphorylated MLKL translocates to the plasma membrane and induces cell death. FPR1, formyl peptide receptor 1; MLKL, mixed lineage kinase domain-like; RIP1, receptor interacting kinase 1.

In 2014, we reported that necroptosis induced by annexin A1–formyl peptide receptor 1 (FPR1) interaction contributes to keratinocyte death in SJS/TEN ^92^. Necroptosis is a type of programmed cell death which reveals morphological necrosis. Necroptotic cells release damage-associated molecular patterns (DAMPs), including a range of pro-inflammatory cytokines, resulting in inflammation, unlike apoptosis. Apoptotic cells are quickly phagocytosed by macrophages and degraded within phagolysosomes. No inflammatory reaction occurs with the process of apoptosis or with the removal of apoptotic cells ^93^. In general, necroptosis occurs through the stimulation of TNF-α under conditions in which apoptosis is blocked. In TNF-α stimulation, receptor interacting kinase 1 (RIP1) and receptor interacting kinase 3 (RIP3) are phosphorylated and form a “necrosome” complex. Furthermore, the mixed lineage kinase domain-like (MLKL) pseudokinase is recruited to the necrosome and phosphorylated by RIP3. The phosphorylated MLKL (pMLKL) is localized to the plasma membrane and induces cell death ^93^. Supernatant from PBMCs, which are exposed to the causative drug in SJS/TEN patients, induces the death of SJS/TEN keratinocytes. This cytotoxicity is blocked by necrostatin-1, a specific inhibitor of RIP1. In SJS/TEN skin lesions, keratinocytes express abundant FPR1 and monocytes secrete annexin A1. The interaction of annexin A1 and FPR1 induces necrosome formation ( Figure 2). Inhibition of necroptosis completely prevents SJS/TEN-like responses in a mouse model of SJS/TEN ^92, 94^. Therefore, these results suggest that necroptosis plays an important role in the pathogenesis of SJS/TEN.

### Diagnostic biomarkers

Although SJS/TEN is a severe disease, clinical manifestations of early stage SJS/TEN are occasionally undistinguishable from those of maculopapular exanthema and erythema multiforme. However, useful biomarkers for the diagnosis or the prediction of severity have not been well established. Recently, some researchers discovered useful diagnostic or prognostic biomarkers for SJS/TEN. These biomarkers are now in the research phase and have not been used in the clinic yet.

Wang *et al.* revealed an increased concentration of CCL-27 in the serum of SJS/TEN patients, which correlated with disease activity ^95, 96^. CCL-27 is reported to be associated with cutaneous inflammatory diseases by regulating the trafficking of T cells to the skin ^97^. Tapia *et al.* found CCL-27 was highly expressed in the skin lesions of SJS/TEN patients ^98^. Wang *et al.* hypothesized that CCL-27 is produced by keratinocytes in the skin lesions found in SJS/TEN and released into the circulation.

Su *et al.* reported that interleukin-15 (IL-15) is associated with mortality and severity in SJS/TEN by measuring 28 serological factors using multiplex immunoassay or ELISA ^99^. They also revealed that IL-15 contributes to TEN severity by enhancing NK- and T-cell-mediated responses. IL-15 is known to induce the production of TNF-α and downstream cytokines/chemokines ^100^. The elevation of many cytokines/chemokines in SJS/TEN might be a secondary effect derived from IL-15.

We identified galectin-7 as a diagnostic biomarker using proteomics analysis ^101^. We hypothesized that certain soluble factors could be secreted only by drug-specific lymphocytes in SJS/TEN patients and not in those with a non-severe cutaneous adverse drug reaction. Hence, these soluble factors could be biomarkers for SJS/TEN. PBMCs from patients with SJS/TEN were cultured with the causative drugs and supernatant was collected. The elevated proteins in the supernatant underwent proteomic analysis ^102^. Hama, Nishimura, and colleagues concluded that this method allowed for the identification of new SJS/TEN-specific biomarkers that are not known to be associated with the pathogenesis of this condition.

Very recently, we focused on the mechanisms of epidermal necroptosis and identified serum RIP3 as a key mediator of necroptosis and as a diagnostic and severity marker ^103^. It is reported that the expression of RIP3 increased in cells undergoing necroptosis ^104^. We revealed that the expression of RIP3 increased in necroptotic keratinocytes as well, and the levels of serum RIP3 were high in the acute phase of patients with SJS/TEN. We also indicated that serum RIP3 levels may correlate with disease activity.

## Management

In SJS/TEN patients, the epidermal and mucosal membranes are predominantly affected. However, SJS/TEN can also cause complications in several organs, such as the liver, kidneys, and respiratory tract. Thus, multidisciplinary assessment and early management in a specialized hospital environment are key for improving mortality.

Immediate discontinuation of suspected causative drugs is crucial in the initial management of SJS/TEN. In addition, supportive care including fluid replacement ^105^, nutritional assessment ^106^, pain relief ^107^, and supplemental oxygen is necessary. Since infection from skin detachment is a common complication in SJS/TEN patients and it is associated with the impairment of re-epithelialization and may lead to sepsis, daily skin care should be performed. Antibiotic treatment should be given when cutaneous infection is clinically suspected ^108^.

The optimal therapeutic strategy in SJS/TEN is still controversial ^109^. Although there have been some reports of benefits with the use of systemic corticosteroids, intravenous immunoglobulins (IVIGs), cyclosporine, TNF-α antagonists (infliximab and etanercept), and plasmapheresis (PP) ^110, 111^, evidence for systemic treatment is still insufficient. UK guidelines for the management of SJS/TEN, published in 2016, concluded that withdrawal of the culprit drug and multidisciplinary supportive care are prioritized over systemic treatment because of the lack of evidence to demonstrate the benefits of the latter ^112^. However, in Japanese guidelines for SJS/TEN, published in 2016, systemic treatment is prioritized over supportive care alone. This guideline recommended early initiation of systemic corticosteroid therapy as a first-line treatment. A combination of IVIG or PP therapy is added to systemic corticosteroid therapy if the clinical symptoms are severe or the disease is refractory to systemic corticosteroid alone.

The efficacy of systemic therapy may depend on the disease phase. For example, in the acute phase, immunosuppressive treatments are considered to be suitable since a strong inflammation-like “cytokine storm” occurs in the patient. However, at the peak period during which wide skin detachment develops, strong immunosuppressive treatment may avoid re-epithelization and increase the risk of infection. Previous studies have not considered this point and included all phase results, leading to discrepant results. We introduce each treatment below.

### Systemic corticosteroids

Previous studies revealed that treatment with corticosteroids in SJS/TEN patients increased the risk of infection and overall complications, including higher mortality ^113– 115^. Analyses and systematic reviews have not revealed a survival advantage of systemic corticosteroids ^116– 118^.

However, recent studies suggested a beneficial role for corticosteroid treatment. A European multicenter retrospective study and recent meta-analysis of observational studies showed the beneficial effects of corticosteroids ^110, 119^. An observational study ** reported that the short-term use of high-dose corticosteroids in the early stages of SJS/TEN reduced mortality without increasing the risk of infection ^120^. Since cutaneous infection is the most important point in the use of corticosteroids for SJS/TEN patients, short-term use of corticosteroids, improvement of infection control, and wound management are necessary to decrease the mortality rate.

### IVIG

IVIG has been widely used for patients with SJS/TEN. However, the mechanisms of IVIG treatment remain unknown. While some case reports concluded that IVIG did not confer a beneficial effect in decreasing mortality ^121– 123^, there are some reports which revealed that IVIG had some beneficial effects for patients with SJS/TEN ^124– 128^. In the largest retrospective study in this field, the European Study of Severe Cutaneous Adverse Reactions (EuroSCAR), IVIG did not improve mortality compared with supportive care alone ^119^. However, recent meta-analyses have shown that high-dose IVIG (<2 g/kg) has a beneficial effect in decreasing the mortality of SJS/TEN ^129^. Thus, the use of IVIG for SJS/TEN patients is still controversial. Randomized controlled trials are required.

### Cyclosporine

Cyclosporine, a calcineurin inhibitor, has been reported to have a therapeutic benefit in SJS/TEN. Cyclosporine affects T-lymphocyte-mediated cytotoxicity and inhibits FasL, nuclear factor-kB, and TNF-α ^130^. Some case reports and meta-analyses have shown that cyclosporine treatment improved mortality in SJS/TEN patients ^131– 138^. Gilbert and Scherrer reported that cyclosporine appears to have not only a mortality benefit in the treatment of SJS/TEN but also few side effects ^139^. These data support a potential role for cyclosporine in the treatment of SJS/TEN. However, the number of reported patients is small. Further studies are required to validate the efficacy of cyclosporine.

### Plasmapheresis

Several case series have shown that PP is effective for the treatment of SJS/TEN ^140– 145^. The purpose of PP is to remove pathogenic factors such as a drug, drug metabolites, and disease-induced cytokines/chemokines from the patient’s blood. PP sessions are carried out every other day or daily. PP is a safe treatment and can be performed with few adverse side effects. Although one observational study has concluded PP treatment to be ineffective, the overall survival in this study was 87.5% ^146^. Narita *et al.* reported that PP was effective in TEN patients who were refractory to supportive therapy or systemic corticosteroid therapy and revealed that cytokine serum levels decreased after PP ^147^.

A study suggested a beneficial effect of combined PP and IVIG therapy ^148^. However, another study reported negative results for the combined therapy while treatment with PP alone revealed a good result ^149^. Randomized studies are needed to further define its usefulness.

### Tumor necrosis factor inhibitors

Owing to the skin lesions and blister fluid in SJS/TEN containing high levels of TNF-α ^150, 151^, TNF-α inhibitors such as etanercept and infliximab have been used and, in some cases, beneficial effects have been suggested ^152– 159^. However, only a small number of cases have reported the use of TNF-α inhibitors for SJS/TEN. Additional studies are required to confirm the efficacy of these drugs in the treatment of SJS/TEN.

### Future directions

Rapid withdrawal of the culprit drug and intensive supportive care are the basis of treatment for SJS/TEN. The use of systemic corticosteroids and IVIG is still controversial. However, recently, there has been an increasing number of studies suggesting the efficacy of cyclosporine or TNF-α inhibitors. Accumulating evidence of these treatments is desirable. In addition, the pathogenesis of SJS/TEN has been elucidated. It is hoped that this research will lead to the discovery of new therapeutic targets.

## Conclusions

This review summarizes recent advances in the pathophysiology, diagnosis, and treatment of SJS/TEN. SJS/TEN is a severe disease which has a high mortality rate. However, its diagnostic method and treatment algorithm have not been well established. Further studies to elucidate the pathogenesis of SJS/TEN are needed.
